# The role of ATP-sensitive potassium channels on DMT1-mediated ferrous iron uptake in SK-N-SH cells

**DOI:** 10.1186/1750-1326-7-S1-S6

**Published:** 2012-02-07

**Authors:** Xixun Du, Huamin Xu, Ning Song, Hong Jiang, Junxia Xie

**Affiliations:** 1 Department of Physiology, Shandong Provincial Key Laboratory of Pathogenesis and Prevention of Neurological Disorders and State Key Disciplines: Physiology, Medical College of Qingdao University, Qingdao 266071, China

## Background

Elevated iron accumulation has been reported in the substantia nigra (SN) in Parkinson’s disease (PD). Our previous study observed that increased levels of the iron importer divalent metal transporter 1 (DMT1) were involved in this nigral iron accumulation and dopaminergic neurons loss in PD. The iron transport function of DMT1 is also related to the membrane potential level, which increases with hyperpolarization of the cell membrane. Activation of ATP-sensitive potassium (K_ATP_) channels, which could induce hyperpolarization of nigral dopaminergic neurons, is reported to be involved in the selective loss of these neurons in PD. The present study is to investigate whether activation of K_ATP_ channels could change the iron uptake function of DMT1.

## Methods

DiBAC4(3) and calcein were used to detect membrane potential and ferrous iron influx. Intracellular iron concentration was measured using an inductively coupled plasma (ICP-2) detector. Mitochondrial transmembrane potential (ΔΨm) and reactive oxygen species (ROS) were measured by flow cytometry using rhodamine123 and H_2_DCF-DA.

## Results

(1) When treated with diazoxide, a novel K_ATP_ channel opener, the membrane potential of SK-N-SH cells showed hyperpolarization. (2) The influx of ferrous iron and the intracellular iron levels were observed dramatically increased when the cells were co-incubated with diazoxide and ferrous iron, resulting in a decreased ΔΨm and an elevated level of ROS production. (3) When treated with diazoxide, cells with DMT1 knockdown showed decreased ferrous iron influx compared with the vector control. (4) When treated with diazoxide, cells overexpressed of SUR1 and Kir6.2 showed increased ferrous iron influx compared with the vector control, which induced a decrease in ΔΨm and an increase in ROS production.

## Conclusion

These results suggest that the activation of the K_ATP_ channels could enhance DMT1-mediated ferrous iron uptake, leading to increase intracellular oxidative stress.

